# Examining the diagnostic value of the mnemonic discrimination task for classification of cognitive status and amyloid-beta burden

**DOI:** 10.1016/j.neuropsychologia.2023.108727

**Published:** 2023-11-07

**Authors:** Soyun Kim, Jenna N. Adams, Miranda G. Chappel-Farley, David Keator, John Janecek, Lisa Taylor, Abanoub Mikhail, Martina Hollearn, Liv McMillan, Paul Rapp, Michael A. Yassa

**Affiliations:** a Department of Neurobiology and Behavior, University of California, Irvine, CA, USA; b Center for the Neurobiology of Learning and Memory, University of California, Irvine, CA, USA; c Department of Psychiatry and Behavioral Sciences, University of California, Irvine, CA, USA; d Department of Military & Emergency Medicine, Uniformed Services University, Bethesda, MD, USA

**Keywords:** Mnemonic discrimination tasks, Alzheimer’s disease, Mild cognitive impairment, Amyloid-beta pathology, Random forest classification, Pattern separation

## Abstract

Alzheimer’s disease (AD) is the most common type of dementia, characterized by early memory impairments and gradual worsening of daily functions. AD-related pathology, such as amyloid-beta (Aβ) plaques, begins to accumulate many years before the onset of clinical symptoms. Predicting risk for AD via related pathology is critical as the preclinical stage could serve as a therapeutic time window, allowing for early management of the disease and reducing health and economic costs. Current methods for detecting AD pathology, however, are often expensive and invasive, limiting wide and easy access to a clinical setting. A non-invasive, cost-efficient platform, such as computerized cognitive tests, could be potentially useful to identify at-risk individuals as early as possible. In this study, we examined the diagnostic value of an episodic memory task, the mnemonic discrimination task (MDT), for predicting risk of cognitive impairment or Aβ burden. We constructed a random forest classification algorithm, utilizing MDT performance metrics and various neuropsychological test scores as input features, and assessed model performance using area under the curve (AUC). Models based on MDT performance metrics achieved classification results with an AUC of 0.83 for cognitive status and an AUC of 0.64 for Aβ status. Our findings suggest that mnemonic discrimination function may be a useful predictor of progression to prodromal AD or increased risk of Aβ load, which could be a cost-efficient, noninvasive cognitive testing solution for potentially wide-scale assessment of AD pathological and cognitive risk.

## 1. Introduction

Alzheimer’s disease (AD) is an age-related neurodegenerative disorder associated with profound cognitive and functional impairment. As the life span of aging population increases, the number of AD cases is projected to reach up to 13.8 million by 2050 in the United States (Hebert et al., 2013) and 152 million worldwide (Zeisel et al., 2020). Currently, there are only a few preventive medications approved (e.g., lecanemab) (van Dyck et al., 2023), imposing a substantial burden on affected individuals, communities, and healthcare systems. AD starts with accumulation of neuropathology many years before symptoms appear (Jack et al., 2013), and about a third of cognitively normal older individuals are known to have high amyloid-beta (Aβ) pathology prior to showing clinical symptoms (Aizenstein et al., 2008; Villemagne et al., 2008). Up to 70% of older individuals who are diagnosed with mild cognitive impairment (MCI), a prodromal stage between normal aging and AD with increased risk for AD dementia, have been shown to have concomitant AD-related pathology (Jansen et al., 2015; Petersen et al., 2001). Because not all the AD-related neuropathology is reversible, early identification and treatment of individuals with high risk for developing MCI or AD is of paramount importance. Timely identification of at-risk individuals will allow early management and planning of long-term care (Leifer, 2003). Prognostics can also help design clinical trials (i.e., screening for target population), facilitate precise intervention plans, and further provide a critical time window for attenuating AD-related symptoms.

Post-mortem neurohistology has been the gold standard for detecting AD pathology including Aβ plaques and neurofibrillary tangles (Hyman and Tanzi, 1992), demonstrating that Aβ deposition starts in the neocortex and gradually progresses toward the allocortex, midbrain, and eventually the cerebellum (Thal et al., 2002). Hyperphosphorylated tau accumulation begins at the locus coeruleus (Ehrenberg et al., 2017; Jacobs et al., 2021), followed by a typical cortical spatiotemporal distribution pattern from the transentorhinal region to the association and sensory cortices (Arnold et al., 1991; Braak and Braak, 1991). A growing volume of work has demonstrated the diagnostic utility of *in vivo* pathological biomarkers to predict AD risk prior to clinical onset using positron emission tomography (PET) imaging (Jack and Holtzman, 2013; Sperling et al., 2011). However, the relationship between the extent of pathology, particularly Aβ, and the severity of cognitive impairment remains debated (Arriagada et al., 1992; Hyman et al., 1993; Ingelsson et al., 2004; Perez-Nievas et al., 2013). For example, a recent meta-analysis by Ackley et al. (2021) analyzing results from 14 randomized controlled trials of Aβ-targeting drugs suggest that these therapies have not yielded meaningful improvements on mini-mental state examination (MMSE) scores. A potentially viable strategy for future trials could be to identify asymptomatic individuals with elevated Aβ burden.

Several *in vivo* tools for measuring AD-related pathology and neurodegeneration, including magnetic resonance imaging (MRI), PET, and fluid biomarkers (e.g., cerebrospinal fluid [CSF], blood, saliva), have opened new research avenues for early detection of pathology and characterizing the AD trajectory. Elevated Aβ and tau measured by either PET or CSF (Quigley et al., 2011; Stephan et al., 2012), brain atrophy (Pini et al., 2016), and functional network dysfunction (Ibrahim et al., 2021; Puttaert et al., 2020) are associated with cognitive status along the AD continuum. While these methods can be useful for early diagnosis and monitoring disease progression, they may be somewhat invasive (e.g., injection of radioactive tracers for PET, or lumbar puncture for CSF collection), expensive, and require a trained expert to analyze and interpret results. Computer-based cognitive testing may provide a low-cost, accessible, and non-invasive solution to early detection of cognitive change and accumulation of key AD pathological markers.

While computerized cognitive testing may be a viable solution to early disease detection, as they can be easily administered during routine clinical assessments in older populations, it is currently unclear what kind of cognitive test may be sensitive and specific enough to detect the earliest pathophysiology or symptoms in AD. Deficits in episodic memory is one of the key hallmarks of AD, and cognitively normal older adults were shown to exhibit deficits in mnemonic discrimination—the ability to differentiate between highly similar objects or events—while more traditional measures of recognition memory remain intact (Berron et al., 2018; Stark et al., 2013; Yassa et al., 2011). This suggests that early impairments in this domain may be clinically meaningful. Early evidence of the neural correlates supporting this function comes from studies of amnesic patients by the late Andrew Mayes and colleagues (Holdstock et al., 2002; Mayes et al., 2002; Migo et al., 2009) who demonstrated that the hippocampus and adjacent cortices play a role in recollection as well as recognition of items that are highly similar (i.e., discrimination) – a major inspiration for the current work. Following Mayes’ pioneering work, a host of studies also have demonstrated impaired recognition and mnemonic discrimination in MCI patients (Belliart-Guerin and Planche, 2023; Bennett et al., 2019; Stark et al., 2013; Yassa et al., 2010).

Evidence from rodent and neuroimaging studies also supported the notion that subfields of the hippocampus (dentate gyrus and CA3) orthogonalize similar patterns into distinct neural representation (i.e., pattern separation), putatively supporting the mnemonic discrimination function (Bakker et al., 2008; Leutgeb et al., 2004). Multiple variants of mnemonic discrimination tasks have been developed across different cognitive domains (e.g., object, spatial, temporal, emotion) (Leal and Yassa, 2014; Reagh et al., 2014, 2018; Stark and Stark, 2017), all of which are considered to be linked to hippocampal integrity (Stark et al., 2019). The direct relationship between AD pathology and mnemonic discrimination performance, however, has not yet been fully assessed.

In this study, we aimed to address these unknowns by taking a machine learning approach to examine whether the mnemonic discrimination tasks (MDT) can be utilized for prediction of cognitive status (normal versus MCI) as well as risks for Aβ burden in a sample of extensively phenotyped older adults without an MCI or dementia diagnosis. Interest in developing machine learning-based approaches for AD prognosis or diagnosis is growing, and numerous studies have demonstrated that artificial intelligence approaches facilitate classification of cognitive status across the AD continuum based on multidimensional datasets (e.g., neuroimaging, genetic biomarker, neuropsychological test scores) as input features (Falahati et al., 2014). While prediction of conversion from MCI to AD or from normal to AD have been extensively studied, prediction of transition from normal to MCI has been less explored, and few studies have examined whether cognitive or behavioral assessments improve model performance. Here, we demonstrate the potential of MDTs as an inexpensive, non-invasive platform for early prognosis of prodromal AD or elevated cerebral Aβ burden.

## 2. Materials and methods

### 2.1. Participants

Data from two studies were used: the Biomarker Exploration in Aging, Cognition, and Neurodegeneration (BEACoN) study and the Alzheimer’s Disease Research Center (ADRC) Project 1 at the University of California, Irvine. The BEACoN study is an ongoing study that aims to develop neuroimaging biomarkers for cognitive decline in preclinical AD. Up to 150 cognitively normal older adults (60 years and older) from community are being enrolled, completing a battery of neuropsychological assessments, mnemonic discrimination tasks, and [^18^F]-Florbetapir (FBP) PET to measure Aβ. Normal cognition was defined as a Clinical Dementia Rating of 0, mini mental state examination (MMSE) score of 27 or higher, and neuropsychological test performance within 1.5 SD of age-adjusted norms.

Project 1 of the ADRC is a completed study originally aimed to recruit cognitively normal older adults (n = 30) as well as older adults with amnestic MCI (n = 15)(Albert et al., 2011) to understand the neural basis of preclinical AD through identification of non-invasive biomarkers. Participants were characterized by the Uniform Data Set (UDS) in accordance with the National Alzheimer’s Coordinating Center (NACC) criteria (Besser et al., 2018; Weintraub et al., 2018) and had a clinician diagnosis (normal or MCI) on cognitive status based on the NACC-UDS guidelines. For MCI, a panel consensus diagnosis was made when 1) there was concern about a change in cognition (by the subject, informant, or clinicians) compared to the subject’s previous level, 2) there was impairment in one or more cognitive domains (memory, executive function, language, attention, and visuospatial skills), and 3) functional independence was preserved for most daily abilities, requiring minimal assistance in living. Participants in the Project 1 also completed the mnemonic discrimination tasks as well as lumbar puncture for cerebrospinal fluid Aβ measurement (see below).

In order to maximize our sample size, we combined data from both studies, ensuring every participant had complete data available including demographic information, amyloid measures (PET or CSF), and performance scores on MDT, MMSE, and Rey auditory verbal learning test (RAVLT). We analyzed a total of 104 participants (mean age = 72.1 yrs, range 60–89 yrs, 65 females), 82 of whom were part of the BEACoN study and 9 of whom had a clinician diagnosis of MCI. All participants were free of major neurological and psychiatric disorders, spoke fluent English, had visual and auditory acuity adequate for neuropsychological and computerized testing, and were free of neuroimaging (MRI or PET) contraindications. Participants gave written informed consent in accordance with the Institutional Review Board of the University of California, Irvine, and were compensated for their participation. Table 1 summarizes the demographic and clinical characteristics of the participants involved in the study.

### 2.2. Aβ cerebrospinal fluid marker

Cerebrospinal fluid samples were collected from the participants in the ADRC cohort via lumbar puncture for analysis of variables related to Aβ load (e.g., Aβ_1–40_, Aβ_1–42_). Following standard clinical research methods in aseptic fashion by a board-certified neurologist, samples were collected in a 15 mL Falcon tube and were placed on ice until processed (within 2 h), aliquoted into 250 μL volumes and stored at −80 °C. The LUMIPULSE *G* 1200, a fully automated immunoassay instrument (Fujirebio, Malvern PA), was used to estimate the levels of Aβ_1–42_ and Aβ_1–40_ markers using a chemiluminescent enzyme immunoassay (CLEIA) by the UCSD Shirley-Marcos ADRC Biomarker Core. The primary measure used for current study was the ratio of Aβ_1–42_ and Aβ_1–40_, accounting for individual differences among Aβ isoforms. A cutoff value of 0.062 (Alcolea et al., 2019) was used to group the participants into two classes: low (>0.062) and high Aβ burden (<0.062).

### 2.3. Amyloid PET image acquisition and processing

Participants in the BEACoN study underwent FBP PET imaging conducted on a High Resolution Research Tomograph at the University of California, Irvine Neuroscience Imaging Center. Image acquisition followed the Alzheimer’s Disease Neuroimaging Initiative (ADNI) (Landau et al., 2013) protocol consisting of 4 × 5-min frames collected 50–70 min after ligand injection. Ten mCi (370MBq) of FBP was injected, followed by a saline flush. During the uptake participants rested comfortably in a dimly lit room with their eyes open.

Structural T1-weighted magnetization prepared rapid gradient echo (MPRAGE) scans were acquired on a 3-T Siemens Magnetom Prisma scanner at the Facility for Imaging and Brain Research, University of California, Irvine, using the following parameters: orientation = sagittal, TR = 2300ms, TE = 2.38ms, FA = 8, voxel resolution = 0.8 mm isotropic, FOV = 256 mm, SENSE acceleration factor = 3. Segmentations were then computed on these images using the Desikan/Killiany atlas in the Freesurfer (Version 6)(Desikan et al., 2006).

The PET data were reconstructed with attenuation correction, scatter correction, and 2 mm^3^ Gaussian smoothing. Images were realigned, co-registered to T1-MPRAGE scans, and normalized by a whole cerebellum reference region to create standard uptake values ratios (SUVR) images. Additional 6 mm^3^ Gaussian smoothing was performed to achieve an effective resolution of 8 mm^3^. The mean SUVR of previously validated cortical composite regions was used to classify the participants into two groups using a cutoff SUVR of 1.10: low (≤1.10) and high (>1.10) Aβ (Landau et al., 2012, 2013).

### 2.4. Concordance between FBP-PET and CSF Aβ measurement

A recent study by Adams et al. (2023) assessed concordance between CSF-based and PET-based Aβ measures with respect to diagnosis of cognition (normal and MCI). The authors showed high concordance between the two measures with high accuracy (0.93 ± 0.13), precision (0.93 ± 0.17), and an overall AUC of 0.92 (±0.16). In the current study, we combined data from both groups for all the analyses.

### 2.5. Rey auditory verbal learning test

The Rey auditory verbal learning test (RAVLT) is a standard neuropsychological verbal memory test that had been widely used as a proxy for episodic memory functions and impairments in aging and dementia (Savage and Gouvier, 1992). The test involves a free recall paradigm in which participants listen to a list of 15 nouns (list A) and are asked to recall aloud as many words as possible. Following 5 repetitions of word recall (A1 to A5), a new list (B) is introduced for “interference” with words presented in the list A. Participants then are asked to recall the words from list A (immediate recall, A6) and 20-mins later asked again to recall as many words as possible (delayed recall, A7). Performance scores were calculated based on the number of words recalled. Other performance metrics were calculated as following: learning rate (learning slope [LS] = [A5–A1]/4); susceptibility to interference (retrospective interference [RI] = A6/A5); forgetting rate ([A5–A7]/5). All the five variables were included as input features.

### 2.6. Mnemonic discrimination tasks

**Fig. 1.**
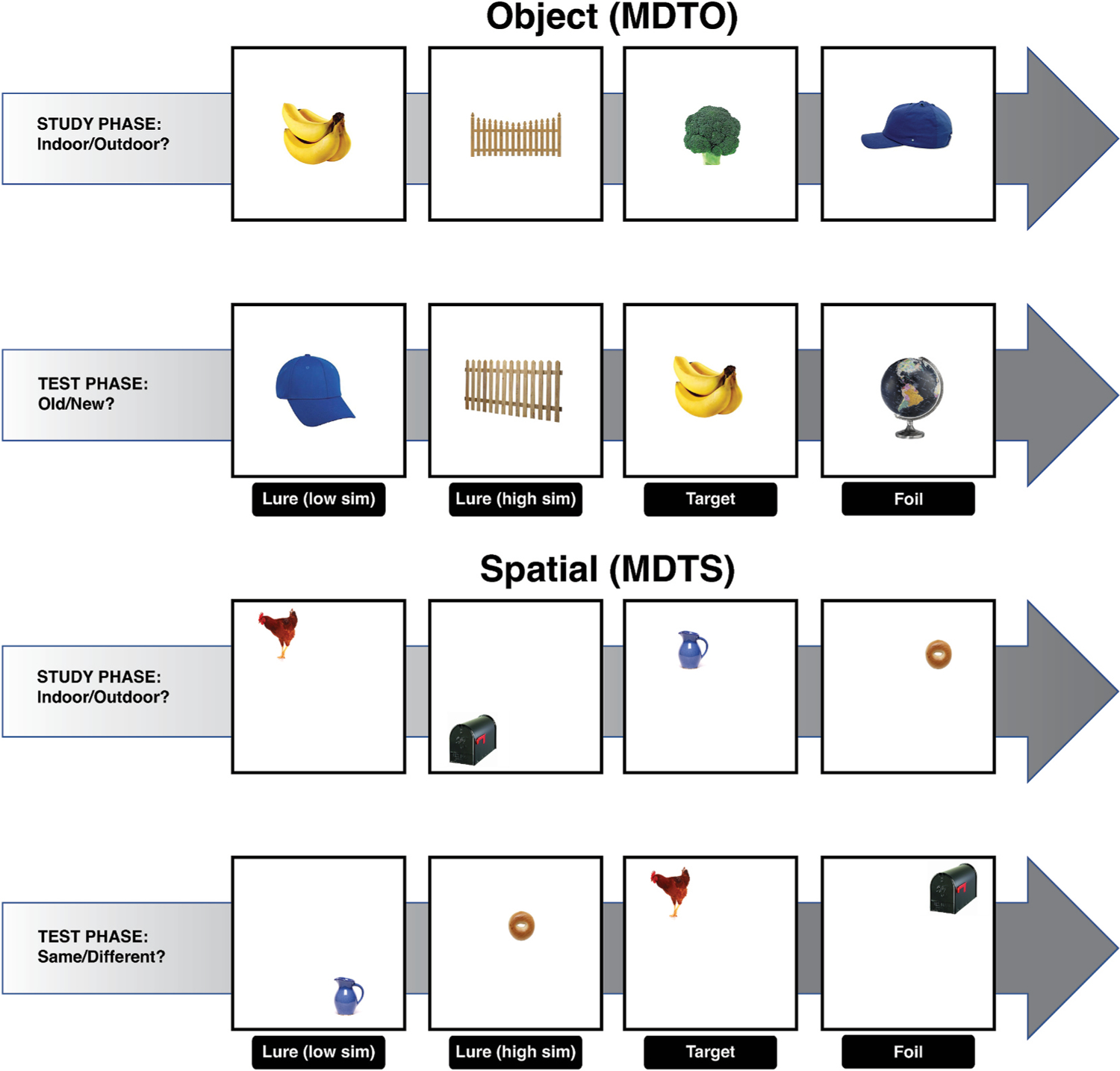
The mnemonic discrimination tasks (MDTs). An illustrative diagram of MDTs for object (MDTO) and spatial (MDTS) domains. Object stimuli size relative to screen size is smaller in the actual task. For each trial, an object image was presented for 2 s (ISI = 0.5sec) in the center of the screen (MDTO) or in a random grid position within the screen (MDTS). During the study phase, participants saw a series of object stimuli and were asked to indicate whether an item belonged “indoor” or “outdoor” objects using a key press. For the test phase, participants were asked to indicate by a keypress whether an object (MDTO) or object location (MDTS) was the “same” or “different”. Participants were allowed 2 s to make a response before the next stimulus appeared. Each participant saw a unique order of stimuli for each phase.

Participants completed the computerized Mnemonic Discrimination Task (MDT) in object (MDTO) and spatial (MDTS) domains, as described previously (Adams et al., 2022; Reagh et al., 2016; Reagh and Yassa, 2014)(Fig. 1). Programmed in Python (version 2.7) using PsychoPy (Peirce, 2007), the MDTs are based on a recognition memory test paradigm including a study (encoding) phase immediately followed by a test (a surprise recognition memory test) phase. During the study phase, participants saw a series of object stimuli (120 for MDTO and 160 for MDTS), one at a time (2 s each, ISI = 0.5 s), on a white background. For each trial, participants were asked to indicate whether an item was an “indoor” or “outdoor” object using a key press. In the MDTO, each object was presented at the center of the screen. In the MDTS, each object was presented in a random grid position within the screen. During the test phase, participants were shown another series of stimuli, one at a time (2 s each, ISI = 0.5 s). For the MDTO, a total of 160 stimuli were shown, including 40 repeated (target) items, 40 new (foil) items, and 80 lure items that were perceptually similar (but not identical) to the studied items. Of the 80 lure items, 40 lures were very similar to the targets (high lures) and 40 lures were less similar to the targets (low lures). Participants were asked to indicate by a keypress whether an object was the “same” or “different”. For the MDTS, the same images were shown as in the study phase, 40 of which were presented in the same locations (targets) and 120 of which were presented in different locations. The distance from the initial presentation varied across incremental levels to manipulate spatial similarity (i.e., novel location for corner-to-corner dislocation, low similarity for positions in a different quadrant of the grid, high similarity for different position within the same quadrant). Participants were asked to indicate by a keypress whether an object was presented in the “same” or “different” position. Participants were allowed 2 s to make a response before the next stimulus appeared. Each participant saw a unique order of stimuli for each phase for both versions.

For both MDTO and MDTS, mnemonic discrimination performance was assessed using a bias-corrected Lure Discrimination Index (LDI), defined as the proportion of lures correctly identified minus the proportion of targets incorrectly identified (p[‘Different’|lures]-p [‘Different’| targets]). We averaged values from LDI of high and low lures. We also calculated LDI as a function of similarity levels (i.e., LDI slope = LDI for low lures minus LDI for high lures). D prime measures, based on classic signal detection theory (Snodgrass and Corwin, 1988), were also calculated for targets, high lures, and low lures based on the equation, d’ = Z (Target hit rate) – Z (False alarm rate). Recognition memory performance was calculated as the probability of correctly responding to repeated target objects (hits) minus the probability of incorrectly responding to foil objects (false alarms). All the five variables were included as input features.

### 2.7. Random forest classification

Random forest (RF) classification models were constructed for prediction of cognitive status (cognitively normal or MCI) and Aβ status (low or high, in cognitively normal participants). Random forest (Breiman, 2001) is a type of ensemble machine learning algorithm that has been widely used for classification or regression models (Fernandez-Delgado et al., 2014). We chose this method based on several advantages in terms of high accuracy, resistance to overfitting and outliers, and robust handling of small samples with class imbalance (Caruana and Niculescu-Mizil, 2006; Fernandez-Delgado et al., 2014). Owing to its utility, random forest methods have been applied in AD studies using large scale data sets such as ADNI (Dimitriadis et al., 2018). An RF classifier selects a random sample of the training data and forms numerous decision trees that learn decision rules from multiple features of the data. Then in a test dataset, prediction of each tree generates the mode of the outcome classes of interest.

We developed and implemented RF algorithms in Python (version 3.8.8) using the Scikit-Learn library (Pedregosa et al., 2011) and built 100 decision trees consisting of different combinations of predictor variables. The predictors or input features consisted of performance scores from MDT, RAVLT, or MMSE. Demographic features (age, sex, education) were also considered in some prediction models. Additionally, we utilized the Gini impurity index (Breiman, 2001), a commonly employed feature selection strategy in RF classification, to assess relative importance of features contributing to model prediction.

Sensitivity and specificity of the models were determined using 0.5 as a threshold. Model performance was then evaluated using the area under the curve (AUC) of the receiver operating characteristic (ROC) curves, which was plotted from all the predicted values of the test data set using leave-one-out cross validation (LOOCV). We specifically chose the LOOCV design given the small sample size (Falahati et al., 2014) and built *n* classification models using *n-1* subject each time and then used the classifier to determine the class of the left out subject. We then performed bootstrapping method (1000 resampling with replacement) to estimate 95% confidence intervals (CI) of the average cross-validated AUCs. DeLong’s method was used to test for significant differences between the AUCs of two models (Delong et al., 1988; Sun and Xu, 2014).

### 2.8. Statistical analysis

All statistical analyses were performed using the open-source statistical software package R (www.r-project.org/). DeLong’s test was performed using the ‘pROC’ package in R. We ran independent two sample t-tests for comparison of demographics and neuropsychological variables between CN and MCI as well as between high Aβ and low Aβ groups. Pearson’s correlation between MDTO, MDTS, and RAVLT was computed to assess collinearity among predictors. Area under the ROC curves were estimated and plotted using Scikit-Learn library implemented in Python (version 3.8.8).

## 3. Results

### 3.1. Demographic, neuropsychological, and clinical characteristics

Participant characteristics are summarized in Table 1, and different types of scores of MDTs are summarized in Supplemental Table 1. Average age (yrs ± SD) of cognitively normal (CN) participants and participants with MCI diagnosis was 71.7 ± 6.6 and 76.5 ± 9.6, respectively. There was a significant difference in age (*p* = 0.05) but not in education level (*p* = 0.62) between the CN and MCI groups. In both groups, there were more females than males and participants were predominantly non-Hispanic white. The MCI group performed more poorly than the CN group on MMSE, immediate and delayed recall on RAVLT, and most of the MDT performance metrics (*p*s < 0.05). Cognitively normal adults were further divided into two groups based on the level of Aβ. There was no group difference in any measures, except for the learning slope of RAVLT (*p* = 0.05). A few participants reported having a comorbid condition (5 diabetes and 2 heart disease).

### 3.2. Correlations among MDTO and MDTS metrics

Pearson’s correlation coefficients between performance scores of MDTO and MDTS are summarized in Table 2, and scatter plots are shown in Supplemental Fig. 1. Overall, the LDI scores of MDTO and MDTS had the highest correlation (r = 0.452, *p* < 0.001) compared to other pairs of scores. Lure discrimination index of MDTO had significant correlation with MDTS LDI, recognition, and d’ (*p*s < 0.001). Recognition score of MDTO also had a strong correlation with LDI and recognition score of MDTS (*p*s < 0.001). Discrimination performance on high spatial lures was significantly associated with MDTO LDI (*p* < 0.05). Similarly, discrimination performance on low spatial lures had significant association with MDTO performance, mainly the LDI and recognition (*p*s < 0.05). When we applied Holm’s correction (Holm, 1979) for multiple correlations, correlations remained statistically significant between MDTO LDI and MDTS LDI, as well as between the recognition score of MDTS and all the MDTO scores.

### 3.3. Correlations among MDTO and RAVLT metrics

Pearson’s correlation coefficients between performance scores of MDTO and RAVLT are summarized in Table 3, and scatter plots are shown in Supplemental Fig. 2. Lure discrimination index, recognition, and d’ scores of MDTO showed significant correlation with immediate recall score (A6) of RAVLT (*p*s < 0.05). LDI, recognition and d’ scores showed significant correlation with delayed recall score (A7) of RAVLT (*p*s < 0.05). All the scores in MDTO had negative correlation with rate of forgetting the word list in RAVLT; however, only recognition and d’ scores had significant association (*p*s < 0.05). No scores in MDTO were significantly associated with learning capacity of the word list in RAVLT. A6 remained significantly correlated with MDTO LDI and recognition score after applying Holm’s correction method.

### 3.4. Correlations among MDTS and RAVLT metrics

Pearson’s correlation coefficients between performance scores of MDTO and RAVLT are summarized in Table 4, and scatter plots are shown in Supplemental Fig. 3. As in MDTO scores, lure discrimination index, recognition, and d’ scores of MDTS showed significant correlation with immediate recall score (A6) of RAVLT or delayed recall score (A7) (ps < 0.05). All the scores in MDTO had negative correlation with rate of forgetting the word list in RAVLT, however, only LDI and recognition scores had significant association (ps < 0.05). No scores in MDTS were significantly associated with learning capacity of the word list in RAVLT. A6 remained significantly correlated with MDTS recognition score after applying Holm’s correction method.

### 3.5. Model performance on classification of cognitive status

**Fig. 2.**
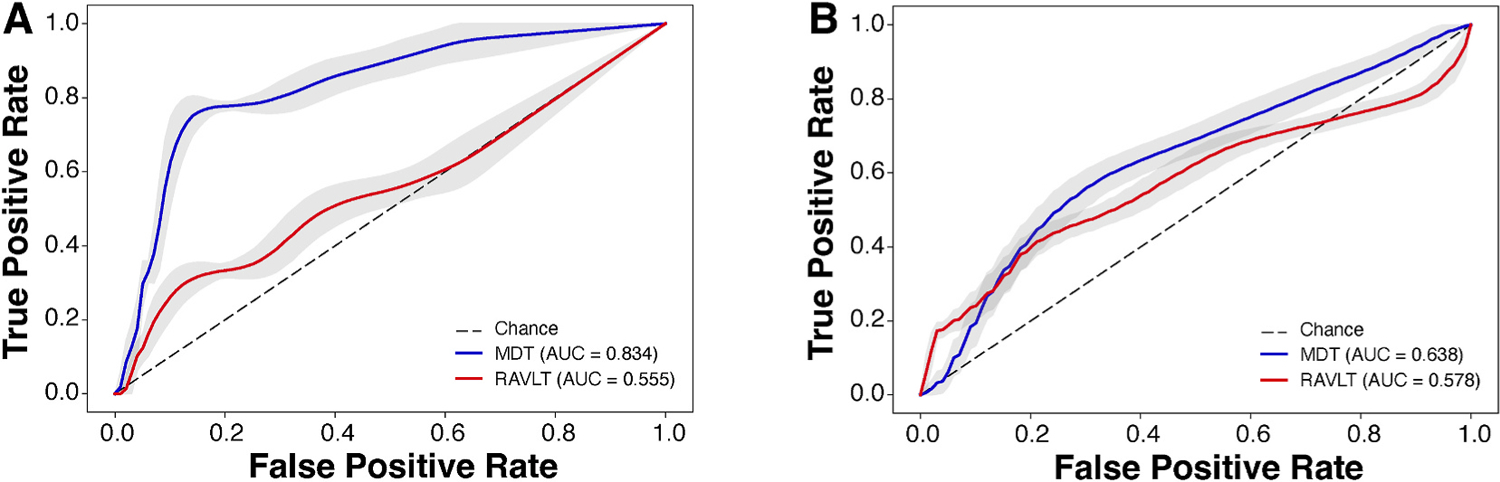
Receiver operating characteristic curves for random forest classification models using MDT performance metrics (blue) and RAVLT performance metrics (red) for cognitive status (A) and amyloid load (B) prediction. MDT = mnemonic discrimination task, RAVLT = Rey auditory verbal learning test, Dotted line = chance.

Table 5 and Fig. 2 demonstrate performance of random forest classification model by area under the curve (AUC) of the ROC curves, specificity, and sensitivity. A prediction model combining 5 types of scores (LDI, target recognition, d’ for target, d’ for high lures, d’ for low lures) from both MDTO and MDTS achieved an AUC of 0.834 (CI 95% bootstrap = 0.833, 0.835) for distinguishing individuals with normal cognition from MCI (Fig. 2A, Table 5). We found weaker performance when employing 5 types of scores (A6, A7, learning rate, retrospective interference, forgetting rate) from RAVLT (AUC = 0.555, CI 95% bootstrap = 0.554, 0.557). The two AUCs were statistically different (DeLong’s test, *p* = 0.01). There was a similar trend when demographics (age, sex, education level) were added to each model (AUC = 0.805 and 0.634 for MDT-based and RAVLT-based predictions, respectively). In fact, the RAVLT-based model performance was weaker than a model predicting with MMSE scores (AUC = 0.716, CI 95% bootstrap = 0.715, 0.717). Models using age, sex, and education level as a single predictor showed weaker performance than models employing MDT scores as predictors (AUCs <0.540).

**Fig. 3.**
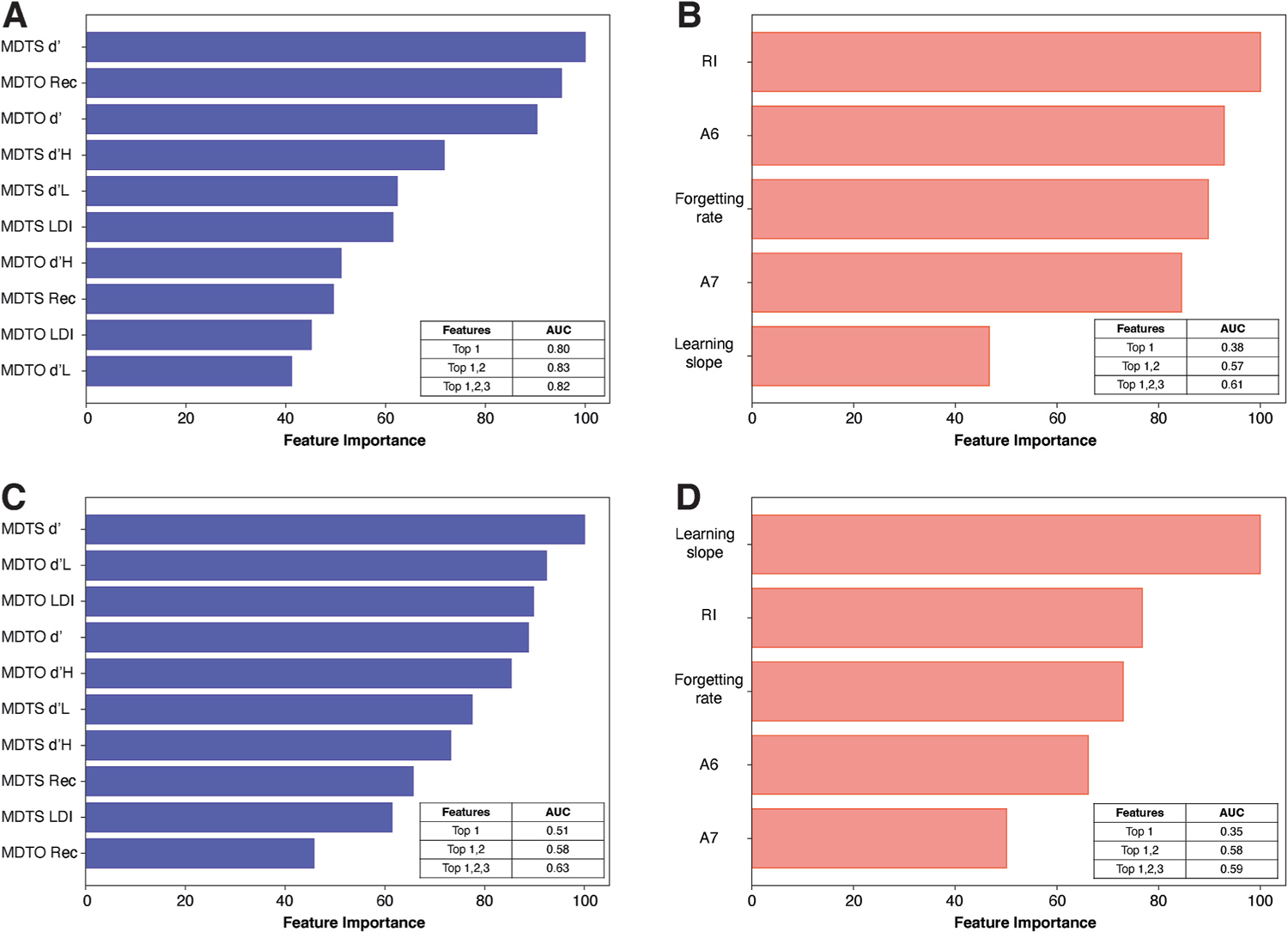
Ranked feature importance among the 10 features of the mnemonic discrimination tasks (A and C) and 5 features of Rey-auditory verbal learning task (B and D) for classification of cognition (A and B) and classification of amyloid status (C and D). Areas under the curve (AUC) for model performance based on the most informative features (up to 3) are tabulated within each plot. MDTO = object version of the mnemonic discrimination task, MDTS = spatial version of the mnemonic discrimination task, Rec = recognition, d’H = d’ for high similarity lures, d’L = d’ for low similarity lures, LDI = lure discrimination index, RI = retrospective interference, A6 = immediate recall, A7 = delayed recall.

Gini impurity-based feature importance was plotted for each model (Fig. 3). The three most informative MDT features were MDTS d’, MDTO recognition, and MDTO d’ (Fig. 3A). For RAVLT, the three most informative features (Fig. 3B) were retrospective interference, immediate recall, and forgetting rate. Feature engineering was performed by selecting the most informative feature (Top 1) and then adding up to three most informative features (Top 1 and 2; Top 1, 2, and 3). For both MDT-based and RAVLT-based models, addition of features enhanced model performance (AUCs tabulated within Fig. 3A and B). On another feature engineering analysis, when recognition scores were excluded, a smaller AUC was achieved (0.79) than the model including all the MDT performance metrics (AUC = 0.83).

To examine whether prediction performance varies between MDT domains (object vs. spatial), we compared model performance of RF classifiers when MDTO and MDTS features were separately employed (Supplemental Figs. 4A–D). A prediction model using MDTO performance metrics achieved an AUC of 0.86, which was numerically higher than the model using both MDTO and MDTS features (AUC = 0.83). This AUC value was higher than that of the MDTS-based model (AUC = 0.75). In the MDTO model, the recognition score emerged as the most informative feature, whereas, in the MDTS model, it was ranked as the least informative.

A joint model combining features from both MDT and RAVLT was considered (Supplemental Fig. 5). The joint model performance did not improve (AUC = 0.81) compared to the MDT-based model with an AUC of 0.83 (Supplemental Fig. 5A). Performance metrics of MDT (e.g., MDTO d’, MDTO Rec, MDTS d’) were ranked as more informative features than RAVLT scores (Supplemental Fig. 5B).

### 3.6. Model performance on classification of Aβ status in cognitively normal older adults

The best model predicting low versus high Aβ burden was achieved for the model in which all the 5 types of scores from both MDTO and MDTS were used ((Fig. 2B, Table 5), yielding an AUC of 0.638 (CI 95% bootstrap = 0.637, 0.639). This performance was superior to that of the model employing the 5 types of scores from RAVLT (AUC = 0.578, CI 95% bootstrap = 0.577, 0.578). The two AUCs were statistically not different (DeLong’s test, *p* = 0.77). When demographics (age, sex, education level) were included as features, MDT-based model (AUC = 0.650, CI 95% bootstrap = 0.649, 0.651) was still superior to RAVLT-based model (AUC = 0.529, CI 95% bootstrap = 0.528, 0.530). Compared to the models employing MDT scores, models employing age (AUC = 0.595, CI 95% bootstrap = 0.594, 0.595), sex (AUC = 0.307, CI 95% bootstrap = 0.307, 0.308), education level (AUC = 0.329, CI 95% bootstrap = 0.326, 0.332), or MMSE scores (AUC = 0.517, CI 95% bootstrap = 0.516, 0.518) as a single predictor demonstrated weaker performance. Interestingly, a model employing the LDI slope as a single feature achieved comparable performance (AUC = 0.648) as the model including all the MDT features and demographics.

Gini impurity-based feature importance was plotted for each model. The three most informative MDT features were MDTS d’, MDTO d’ low similarity lures, and MDTO LDI (Fig. 3C). Recognition scores of MDTS and MDTO were less informative features (8th and 10th, respectively). For RAVLT, the three most informative features were learning slope, retrospective interference, and forgetting rate (Fig. 3D). Feature engineering was performed by selecting the most informative feature (Top 1) and then adding up to three most informative features (Top 1 and 2, Top 1, 2, and 3). For both MDT and RAVLT models, addition of features also enhanced model performance (AUCs tabulated within Fig. 3C and D). On another feature engineering analysis, exclusion of recognition scores had little impact on model performance (AUC = 0.66) compared to the model including all the MDT performance metrics (AUC = 0.64).

To examine if prediction performance varies between MDT domains (object vs. spatial), we compared model performance of RF classifiers when MDTO and MDTS features were separately employed (Supplemental Figs. 4E–H). A prediction model using features from MDTO performance metrics achieved an AUC of 0.63, which was similar to the model using both MDTO and MDTS features (AUC = 0.64). The MDTO-based model yielded a marginally higher AUC than the MDTS-based model (AUC of 0.60). For each MDTO and MDTS model, recognition scores were the least informative feature.

A joint model combining features from MDT and RAVLT was considered (Supplemental Fig. 5). The joint model performance remained consistent (AUC = 0.65) compared to the MDT-based model with an AUC of 0.64 (Supplemental Fig. 5C). Recognition scores of MDT and recall scores (A6 and A7) of RAVLT ranked as the least informative features (Supplemental Fig. 5D).

## 4. Discussion

In this study, we evaluated the diagnostic utility of the mnemonic discrimination tasks for cross-sectionally identifying cognitively normal older individuals from individuals with a clinical diagnosis of MCI, as well as for statistically predicting cerebral Aβ burden (measured by FBP-PET or CSF) among cognitively normal older adults. We trained a random forest classifier by including various MDT performance scores as input features. For comparisons, we also trained random forest classifiers by including performance metrics from a commonly used neuropsychological assessment (RAVLT) or a MMSE score. Model performance was evaluated based on the AUC of ROC curves. For classification of cognitive status, the MDT-based model outperformed the RAVLT-based model with a higher AUC (0.83 vs. 0.56, DeLong’s test, p < 0.05). For classification of Aβ status, the MDT-based model yielded a numerically higher AUC than the RAVLT-based model (0.64 vs. 0.53), but the AUCs were not statistically different (DeLong’s test, *p* > 0.5). For both classifications, MDT recognition scores had a minimal impact on model performance. Furthermore, combining MDT and RAVLT features did not enhance classification compared to models that used MDT performance metrics as features. Overall, RF classifiers based on MDT performance metrics yielded numerically greater AUC values than classifiers based on RAVLT performance metrics.

A key aspect of the MDT is its ability to engage the hippocampal-dependent process (pattern separation) that supports discrimination of highly similar representations. Notably, a body of literature (Aggleton and Shaw, 1996; Holdstock et al., 2005; Mayes et al., 2002) has demonstrated sparing of recognition memory following hippocampal lesions, suggesting that recognition and mnemonic discrimination are two distinct cognitive processes. This idea leads to an interesting question: how might including recognition scores impact the performance of our MDT-based classifiers? In our classifications of cognition or Aβ status, recognition scores had minimal impact on model predictions, resulting in marginal changes in the AUC values. Moreover, recognition scores ranked as less informative features for classification of Aβ load in cognitively normal individuals. These findings are in line with the idea that recognition remains largely intact over healthy aging (e.g.., Stark et al., 2013) and demonstrate that predicting AD-related pathology based on recognition performance may not yield sufficient discrimination.

A line of research exploring the domain-specific (object and spatial) functional pathways in the MTL (Ranganath and Ritchey, 2012) may offer relevant insights into to our results comparing the discriminative value of MDTO and MDTS. These functional pathways operate separately for object information through the perirhinal-lateral entorhinal cortex and spatial information through the parahippocampal-medial entorhinal cortex, which are also thought to be crucial for MDTO and MDTS, respectively (Reagh and Yassa, 2014). Importantly, the transitional region including the lateral entorhinal and perirhinal cortex is known to be vulnerable to accumulation of AD-related pathology (primarily tau pathology, Braak and Braak, 1991), overlapping with the object processing pathway that supports MDTO. In our classification of CN and MCI, we found that that the MDTO-based model achieved a higher AUC value of 0.86, surpassing the model that exclusively used MDTS features (AUC = 0.75). While this result is in line with other reports demonstrating impaired MDTO performance in MCI (Bakker et al., 2012; Stark et al., 2013), there is still lack of evidence supporting which domain serves as the superior tool for clinical diagnosis. We also compared model performance between MDTO and MDTS for prediction of Aβ status but found little difference (AUC = 0.63 vs. 0.60). One possibility is that, because MDTO and MDTS metrics were correlated, it was expected that no specific domain outperforms the other. Another possibility is that the domain difference may track tau pathology better than Aβ, given the susceptibility of the transentorhinal region to the pathology (Berron et al., 2018, 2019; Braak and Braak, 1991), which invites future work examining the domain-specific diagnostic value for prediction of tau burden.

For classification of cognition, we found that the RAVLT-based model marginally benefited with addition of demographic features. We also trained RF classifiers using combined features from both MDT and RAVLT, and this joint model performance substantially improved (AUC = 0.81) compared to the RAVLT-based model (AUC = 0.56). A few points may help explain these findings. First, MCI is recognized as a heterogeneous category, encompassing a wide range of demographics, cognitive or clinical symptoms, and pathological severity. Due to this heterogeneity, we expect the clinical diagnosis may not achieve perfect performance (i.e., AUC close 1.0). In this regard, our MDT-based classifier may have already reached a near-ceiling level of performance (AUC >0.8), and further improvement may not be possible. Second, MDT performance may be more sensitive to age (e.g., decline starting to emerge in the 4th decade of life) than RAVLT performance (Stark et al., 2013). It is plausible that MDT performance already accounts for age-related effects and therefore, addition of age or demographic features may have had little impact on model predictions. Overall, our findings suggest that MDT may be an effective diagnostic tool and able to provide complementary information to cognitive assessments used for diagnosis of AD.

A wealth of studies have utilized machine learning techniques and integrated combined features from biomarkers and neuropsychological scores for discrimination of healthy controls from AD patients (Clark et al., 2014; Ewers et al., 2012; Spasov et al., 2019), for discrimination of MCI from AD (Ardekani et al., 2017; Clark et al., 2016; Grassi et al., 2019; Moradi et al., 2015; Velazquez et al., 2021; Zhang et al., 2011), or for discrimination of normal cognition from MCI (Cui et al., 2012; Gray et al., 2013; Lebedeva et al., 2017). Most of these investigations yielded AUCs ranging from 0.7 to 0.9, suggesting that biomarker data derived from neuroimaging, CSF assays, or genotyping play a crucial role in achieving powerful classification of cognition along the AD continuum. A fundamental question remains whether cognitive test metrics can adequately replace the need of acquiring invasive and expensive biomarker data. While conversion to AD from MCI is a stage that may be readily detectable with standard neuropsychological measures across multiple cognitive domains, conversion from cognitively normal to MCI is perhaps a more subtle and less common trajectory. Consequently, more sensitive and specific cognitive tests are required to predict the transition to MCI, which is a critical window for potential intervention. Our findings illustrate that by probing memory measures (MDT) rooted in neurobiological process (pattern separation) and known to be sensitive to cognitive decline, predicting conversion from cognitively normal to MCI may be achievable.

We also examined whether MDT performance may predict risks of cerebral Aβ burden in older adults with normal cognition. A few studies recently proposed different machine-learning approaches for predicting risks for cerebral Aβ positivity, but mostly in MCI or prodromal AD patients (Ezzati et al., 2020; Haghighi et al., 2015; Kandel et al., 2015; Kim et al., 2018, 2021; Palmqvist et al., 2019) reporting a range of AUCs between 0.6 and 0.8. Our classification performance for Aβ status was generally weaker than classification for clinical diagnosis. One potential explanation is that features like neuroimaging data, APOE genotype, or biomarkers exhibit higher discriminatory power in detecting AD pathology, whereas cognitive impairment alone may not be sufficient to differentiate pathological burden. Another possibility is that Aβ accumulation is prevalent in cognitively normal older adults (Jack and Holtzman, 2013) and is only weakly associated with cognitive changes. In contrast to tau, which tends to be regionally concentrated around the medial temporal lobe in preclinical AD (Braak and Braak, 1991), the distribution of Aβ plaques is widespread throughout the brain (Thal et al., 2002). Therefore, performance on MDT, which taps into medial temporal lobe integrity, may not exhibit a strong association with Aβ pathology. As suggested above, future work is needed to examine the diagnostic value of MDTs for prediction of tau burden.

Though not based on machine-learning approaches, some recent studies (Jutten et al., 2021a; Papp et al., 2021; Trelle et al., 2021; Webb et al., 2020) are worth noting for demonstrating a close relationship between MDT performance and AD pathology in various cognitively normal cohorts. Papp et al. (2021), for example, used a variant of the MDTO (labelled as the Behavioral Pattern Separation Test-Object [BPSO]) and demonstrated significant differences between a large sample (n = 4486) of Aβ + and Aβ – individuals. Aβ or tau burden was also found to be related to lure discrimination scores particularly as a function of similarity level in cognitively normal older adults, a pattern which was observed in both MDTO (Trelle et al., 2021) and MDTS (Webb et al., 2020). While there are methodological and analytical differences among the studies, the results are in line with our finding that MDT performance metrics may be a useful tool for early detection of AD-related pathology in cognitively normal older adults.

With the growing number of large data sets, machine learning has emerged as a useful diagnostic approach in numerous clinical and non-clinical domains. Among several ensemble learning techniques available (Caruana and Niculescu-Mizil, 2006; Fernandez-Delgado et al., 2014), we chose the random forest classifier given its advantages for effective handling of small or imbalanced data and capability of solving overfitting problems (Dietterich, 2000). Another strength of the RF classifier is that relative feature importance can be derived, providing additional explanations for prediction models. In recent review papers, classification results from studies using RF classifiers (Sarica et al., 2017) or other prediction methods (e.g., support vector machine, neural network) (Weiner et al., 2017) for clinical diagnosis of AD have been summarized. A study by Velazquez et al. (2021) particularly provided comparisons among RF classifier, support vector classifier, logistic regression, and XGBoost classifier, and found RF was the best method for AD conversion prediction. Collectively, previous reports demonstrate the utility of RF classifiers as an early screening tool to identify individuals who will likely develop AD and require intervention.

In addition to the use of RF classification algorithms, strengths of our study include our focus on discrimination of CN and MCI in the early stage of AD, which provide opportunities for identifying at-risk individuals prior to any clinical diagnosis. Moreover, our study participants have been deeply phenotyped with regards to their cognitive and clinical profiles. Our study is also among the few studies leveraging rich, comprehensive datasets to examine the diagnostic capacity of cognitive tests for cerebral Aβ status, which can otherwise be measured by invasive and expensive means. The MDT can be briefly and easily administered in several domains (object, spatial, temporal) and in different platforms (in person or online) (Jutten et al., 2021a). Furthermore, compared to RAVLT that primarily rely on verbal memory, the MDT stimuli are based on object images and therefore can be widely available for diverse population speaking different languages (e.g., see Suwabe et al., 2017).

The main limitation of our study is the small and unbalanced sample size (e.g., MCI, n = 9) compared to other studies using large-scale databases (e.g., ADNI cohort). Thus, our statistical power for classification may be somewhat limited and warrants cautious interpretation of the findings. Although we were not able to test our RF classifiers on an independent dataset, we mitigated this issue by using the leave-one-out cross validation that is considered the preferred method for smaller datasets (Falahati et al., 2014). We also note that sensitivity values of our models were quite low (16%–47%) (Table 5). Given the imbalance in the class of the participant groups, we suggest that little importance can be assigned to the divergence of the specificity and sensitivity values. This emphasizes the importance of obtaining confidence intervals for these measures. Consider for example, a study by Wang et al. (2017) which investigated the use of event related potentials to identify individuals at risk of presenting delayed onset post-traumatic stress disorder. The participant groups in this study were unbalanced (60 Stables, 5 Converters). In this case on a first examination the results were encouraging (sensitivity = 0.80, specificity = 0.87), but the confidence intervals for both measures were found to be [0.0, 1.0]. The sensitivity and specificity results were seen to be a fortuitous consequence of the small sample size. Similarly, in the current investigation, the divergence of the sensitivity or specificity results may be a consequence of an unbalanced design.

Another limitation of our study is that our participants were predominantly non-Hispanic white with high educational attainment, which necessitates future studies with a larger and more diverse sample. Also, our participants were generally free of other neuropsychiatric conditions (e.g., depression), which may make our findings less generalizable to the broader aging population. Given the relatively high prevalence of neuropsychiatric disorders in preclinical AD (Ownby et al., 2006), future studies should consider these conditions in their analyses. Lastly, our analyses were cross-sectional, making it challenging to examine how MDT performance may predict subsequent cognitive or neuropathological changes. Future work with longitudinal data will provide much insight into this potential relationship.

## 5. Conclusion

In summary, we have demonstrated that the MDT has a diagnostic utility in classifying AD-related cognitive impairment as well as Aβ burden in a preclinical AD population. Our findings indicate that utilizing cost- and time-efficient digital cognitive assessments can effectively predict cognitive status or AD-related pathology, replacing the need for expensive and invasive measures. This approach can serve as an initial screening tool, guiding clinical decision-making without subjecting individuals to risky and financially burdensome assessments. Furthermore, the machine-learning techniques employed in this research can be extended to other datasets, including diverse ethnic and racial populations. This extension represents a crucial direction for future research, enhancing the applicability and generalizability of the findings.

## Supplementary Material

Supplement

## Figures and Tables

**Table 1:** Demographics and clinical and neuropsychological characteristics of the study participants.

Demographics Total (n = 104)	CN		MCI	Group difference^a^	Group difference^b^
	Low Aβ	High Aβ		p value	p value
**N**	67	28	9	–	–
**Age (yrs)**	71.1 (6.3)	73.1 (7.3)	76.5 (9.6)	0.05	0.19
**Female (%)**	61.2	78.6	22.2	–	–
**Education (yrs)**	16.8 (2.3)	16.1 (2.5)	17.0 (2.3)	0.62	0.17
**Race (%)**					
White	71.6	89.3	88.9	–	–
Asian	22.4	10.7	11.1	–	–
Black	1.5	0	0	–	–
More than one	1.5	0	0	–	–
Other	3.0	0	0	–	–
**MMSE**	28.4 (1.3)	28.6 (1.5)	27.4 (2.1)	0.04	0.34
**MDTO LDI**	0.27 (0.15)	0.31 (0.12)	0.198 (.10)	0.10	0.14
**MDTO Rec**	0.81 (0.15)	0.81 (0.11)	0.64 (0.18)	0.001	0.95
**MDTO d’**	3.58 (1.75)	3.19 (1.45)	2.08 (0.90)	0.02	0.31
**MDTO d’High**	0.87 (0.93)	0.92 (0.62)	0.50 (0.24)	0.18	0.79
**MDTO d’Low**	1.42 (0.94)	1.44 (0.75)	0.91 (0.35)	0.09	0.91
**MDTS LDI**	0.25 (0.16)	0.28 (0.12)	0.09 (0.10)	0.001	0.46
**MDTS Rec**	0.39 (0.21)	0.43 (0.19)	0.11 (0.13)	<0.001	0.42
**MDTS d’**	1.26 (0.90)	1.68 (1.50)	0.31 (0.39)	0.01	0.10
**MDTS d’High**	0.60 (0.71)	0.69 (0.80)	0.11 (0.31)	0.04	0.59
**MDTS d’Low**	1.03 (0.80)	1.27 (1.02)	0.71 (0.99)	0.22	0.22
**RAVLT - immediate**	10.8 (3.1)	11.0 (3.4)	6.7 (3.6)	<0.001	0.79
**RAVLT - delayed**	10.8 (3.3)	10.7 (3.8)	5.8 (4.8)	<0.001	0.85
**RI**	0.84 (0.17)	0.85 (0.20)	0.65 (0.24)	0.004	0.72
**Learning**	1.74 (0.51)	1.49 (0.61)	1.44 (0.78)	0.27	0.05
**Forgetting**	0.17 (0.19)	0.17 (0.24)	0.46 (0.39)	<0.001	0.99
**Comorbidities**					
Diabetes	4	0	1	–	–
Heart disease	2	0	0	–	–

Data are presented as mean (SD). Group differences were assessed by two-sample independent T tests. CN = cognitively normal, MCI = mild cognitive impairment, Aβ = amyloid, MMSE = mini-mental state exam, MDTO = object version of the mnemonic discrimination task, MDTS = spatial version of the mnemonic discrimination task, LDI = lure discrimination index, Rec = recognition, d’High = d’ for high similarity lures, d’Low = d’ for low similarity lures, RAVLT = Rey auditory verbal learning test, RI = retrospective interference.

a CN vs. MCI,

b Low Aβ vs. High Aβ

**Table 2:** Pearson correlation coefficients among MDTO and MDTS scores.

	MDTS LDI	MDTS Rec	MDTS d’	MDTS d’H	MDTS d’L
MDTO LDI	.452*^†^	.425*^†^	.307*^†^	.198*	.217*
MDTO Rec	.337*^†^	.445*^†^	.205*	.102	.197*
MDTO d’	.176	.310*^†^	.112	.001	.063
MDTO d’H	.173	.297*^†^	.155	.062	.022
MDTO d’L	.195	.313*^†^	.171	.082	.058

MDTO = object version of the mnemonic discrimination task, MDTS = spatial version of the mnemonic discrimination task, LDI = lure discrimination index, Rec = recognition, d’H = d’ for high similarity lures, d’L = d’ for low similarity lures,

* significant, uncorrected *p* < 0.05,

† significant, corrected (Holm’s) *p* < 0.05.

**Table 3:** Pearson correlation coefficients among MDTO and RAVLT scores.

	A6	A7	LS	RI	FR
MDTO LDI	.317*^†^	.228*	.048	.280*	−.166
MDTO Rec	.306*^†^	.271*	.005	.273*	−.257*
MDTO d’	.240*	.237*	.072	.196*	−.221*
MDTO d’H	.210*	.182	.055	.195*	−.173
MDTO d’L	.225*	.164	.026	.217*	−.147

MDTO = object version of the mnemonic discrimination task, RAVLT = Rey auditory verbal learning test, LDI = lure discrimination index, Rec = recognition, d’H = d’ for High similarity lures, d’L = d’ for low similarity lures, LS = learning slope, RI = retrospective interference, FR = forgetting rate,

* significant, uncorrected *p* < 0.05,

† significant, corrected (Holm’s) *p* < 0.05.

**Table 4:** Pearson correlation coefficients among MDTS and RAVLT scores.

	A6	A7	LS	RI	FR
MDTS LDI	.295*	.296*	.106	.247*	−.267*
MDTS Rec	.318*^†^	.286*	.091	.252*	−.240*
MDTS d’	.259*	.212*	−.006	.202*	−.163
MDTS d’H	.112	.072	−.034	.110	−.064
MDTS d’L	.096	.053	−.046	.115	.041

MDTS = spatial version of the mnemonic discrimination task, RAVLT = Rey auditory verbal learning test, LDI = lure discrimination index, Rec = recognition, d’H = d’ for High similarity lures, d’L = d’ for low similarity lures, LS = learning slope, RI = retrospective interference, FR = forgetting rate,

* significant, uncorrected *p* < 0.05,

† significant, corrected (Holm’s) *p* < 0.05.

**Table 5:** Model performance evaluation metrics.

Classification	Features	AUC [CI]	Sensitivity (%) [CI]	Specificity (%) [CI]
**Cognitive status (CN vs. MCI)**	MDT	.834 [.833 .835]	34.3 [33.8 34.8]	93.0 [93.0 93.1]
	RAVLT	.555 [.554 .557]	16.6 [15.8 17.5]	91.7 [91.6 91.7]
**Aβ status (Low vs. High)**	MDT	.638 [.637 .639]	47.0 [46.6 47.3]	73.9 [73.8 74.0]
	RAVLT	.578 [.577 .578]	47.1 [46.8 47.4]	74.7 [74.6 74.8]

AUC = area under the curve, CI = 95% confidence intervals, CN = cognitively normal, MCI = mild cognitive impairment, MDT = mnemonic discrimination task, RAVLT = Rey auditory verbal learning test.

## Data Availability

Data will be made available on request.
