# Supplementary material for: Examining the diagnostic value of the mnemonic discrimination task for classification of cognitive status and amyloid-beta burden
Source: Neuropsychologia. Author manuscript; Available in PMC 2024 Jan 3. (PMC10764118; doi:10.1016/j.neuropsychologia.2023.108727)
Supplement: Supplement [file NIHMS1953778-supplement-Supplement.docx]

Supplemental Table 1. Performance scores (mean and sd) obtained by each participant group (low amyloid, high amyloid, MCI) on the mnemonic discrimination tasks

| **Test** | **Scores** | **CN** | | | | **MCI** | |
| --- | --- | --- | --- | --- | --- | --- | --- |
|  |  | **Low amyloid**  **(n = 67)** | | **High amyloid**  **(n = 28)** | | **(n = 9)** | |
|  |  | mean | sd | mean | sd | mean | sd |
| **MDTO** | Target Correct | 0.89 | 0.10 | 0.89 | 0.07 | 0.84 | 0.11 |
|  | Target Incorrect | 0.11 | 0.10 | 0.11 | 0.07 | 0.16 | 0.11 |
|  | Lure High Correct | 0.28 | 0.14 | 0.33 | 0.15 | 0.29 | 0.16 |
|  | Lure High Incorrect | 0.72 | 0.14 | 0.67 | 0.15 | 0.71 | 0.16 |
|  | Lure Low Correct | 0.47 | 0.18 | 0.51 | 0.16 | 0.43 | 0.19 |
|  | Lure Low Incorrect | 0.53 | 0.18 | 0.49 | 0.16 | 0.57 | 0.19 |
|  | Foil  Correct | 0.92 | 0.10 | 0.92 | 0.07 | 0.80 | 0.13 |
|  | Foil  Incorrect | 0.08 | 0.10 | 0.08 | 0.07 | 0.20 | 0.13 |
| **MDTS** | Target Correct | 0.63 | 0.17 | 0.66 | 0.15 | 0.50 | 0.19 |
|  | Target Incorrect | 0.37 | 0.17 | 0.34 | 0.15 | 0.50 | 0.19 |
|  | Lure High Correct | 0.55 | 0.17 | 0.54 | 0.20 | 0.55 | 0.18 |
|  | Lure High Incorrect | 0.45 | 0.17 | 0.46 | 0.20 | 0.45 | 0.18 |
|  | Lure Low Correct | 0.70 | 0.18 | 0.71 | 0.16 | 0.63 | 0.22 |
|  | Lure Low Incorrect | 0.30 | 0.18 | 0.29 | 0.16 | 0.37 | 0.22 |
|  | Foil  Correct | 0.76 | 0.18 | 0.77 | 0.15 | 0.61 | 0.21 |
|  | Foil  Incorrect | 0.24 | 0.18 | 0.23 | 0.15 | 0.39 | 0.21 |

CN = cognitively normal, MCI = mild cognitive impairment, MDTO = object version of the mnemonic discrimination task, MDTS = spatial version of the mnemonic discrimination task, sd = standard deviation.


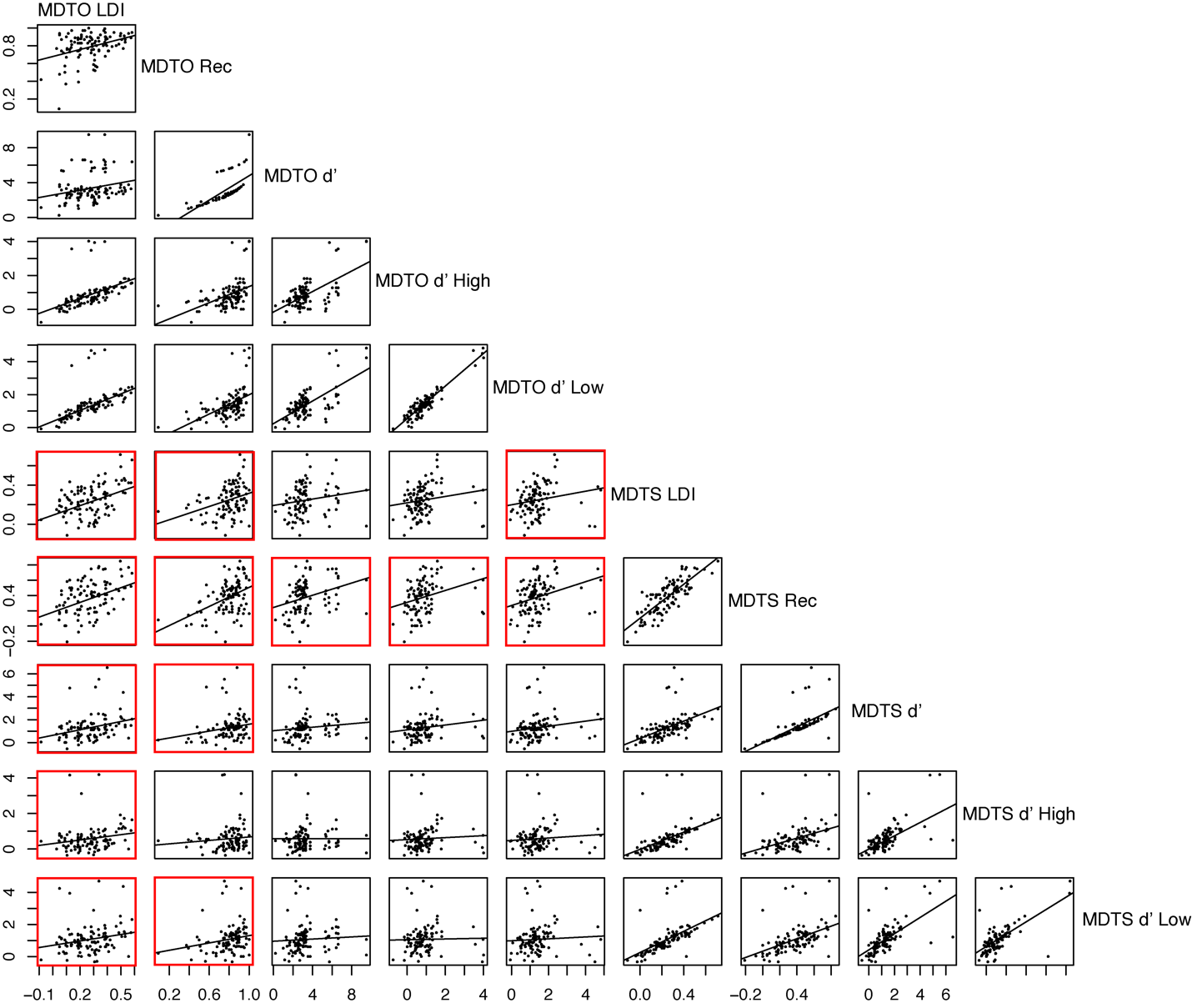


Supplemental Fig 1. Scatter plots illustrating Pearson’s correlations between performance metrics between the mnemonic discrimination tasks. Significant correlations (uncorrected p < 0.05) are indicated in red. MDTO = object version of the mnemonic discrimination task, MDTS = spatial version of the mnemonic discrimination task, LDI = lure discrimination index, Rec = recognition, d’High = d’ for high similarity lures, d’Low = d’ for low similarity lures.


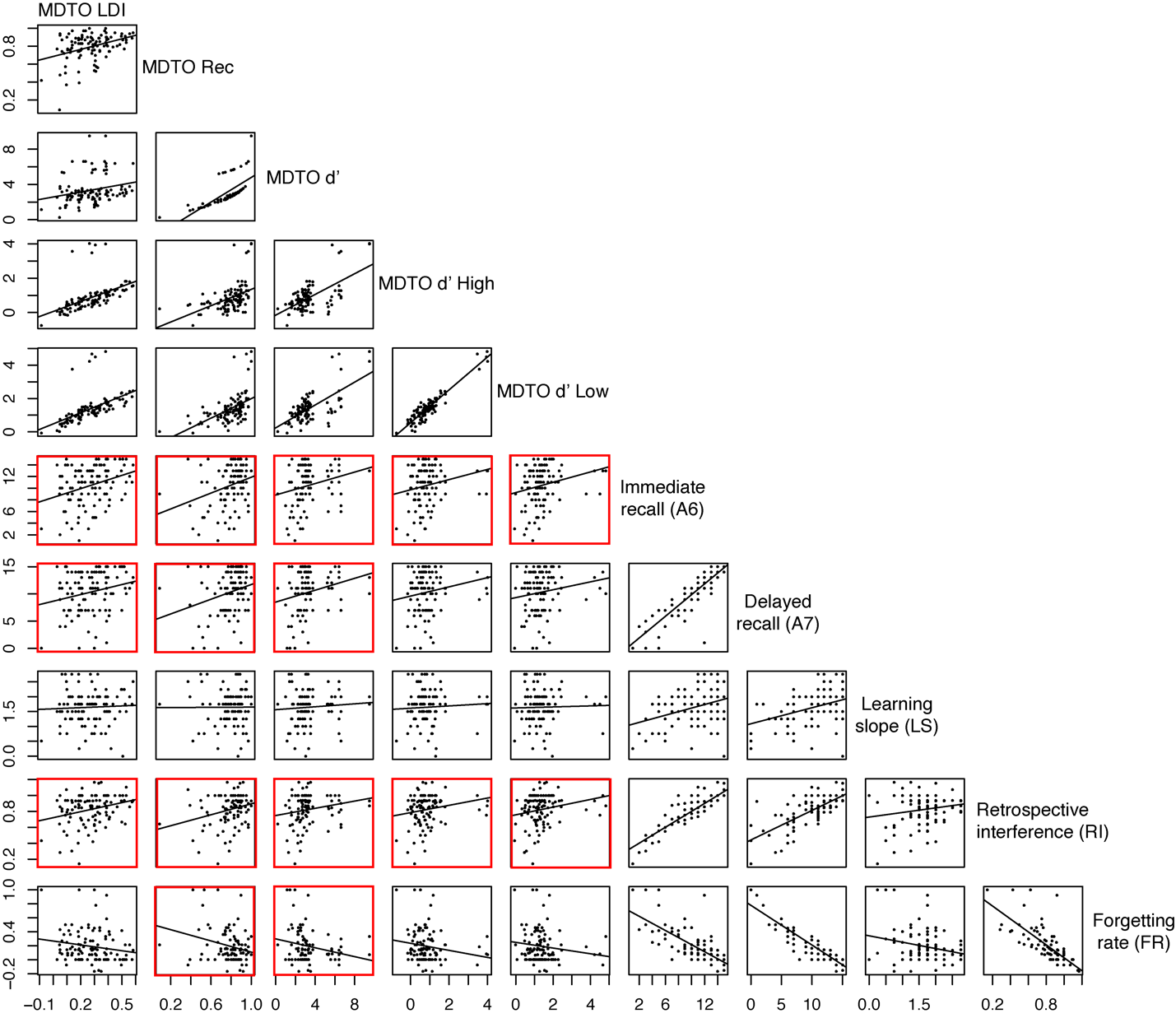


Supplemental Fig 2. Scatter plots illustrating Pearson’s correlations between performance metrics between the object version of the mnemonic discrimination task (MDTO) and Rey auditory verbal learning test. Significant correlations (uncorrected p < 0.05) are indicated in red. LDI = lure discrimination index, Rec = recognition, d’High = d’ for high similarity lures, d’Low = d’ for low similarity lures.


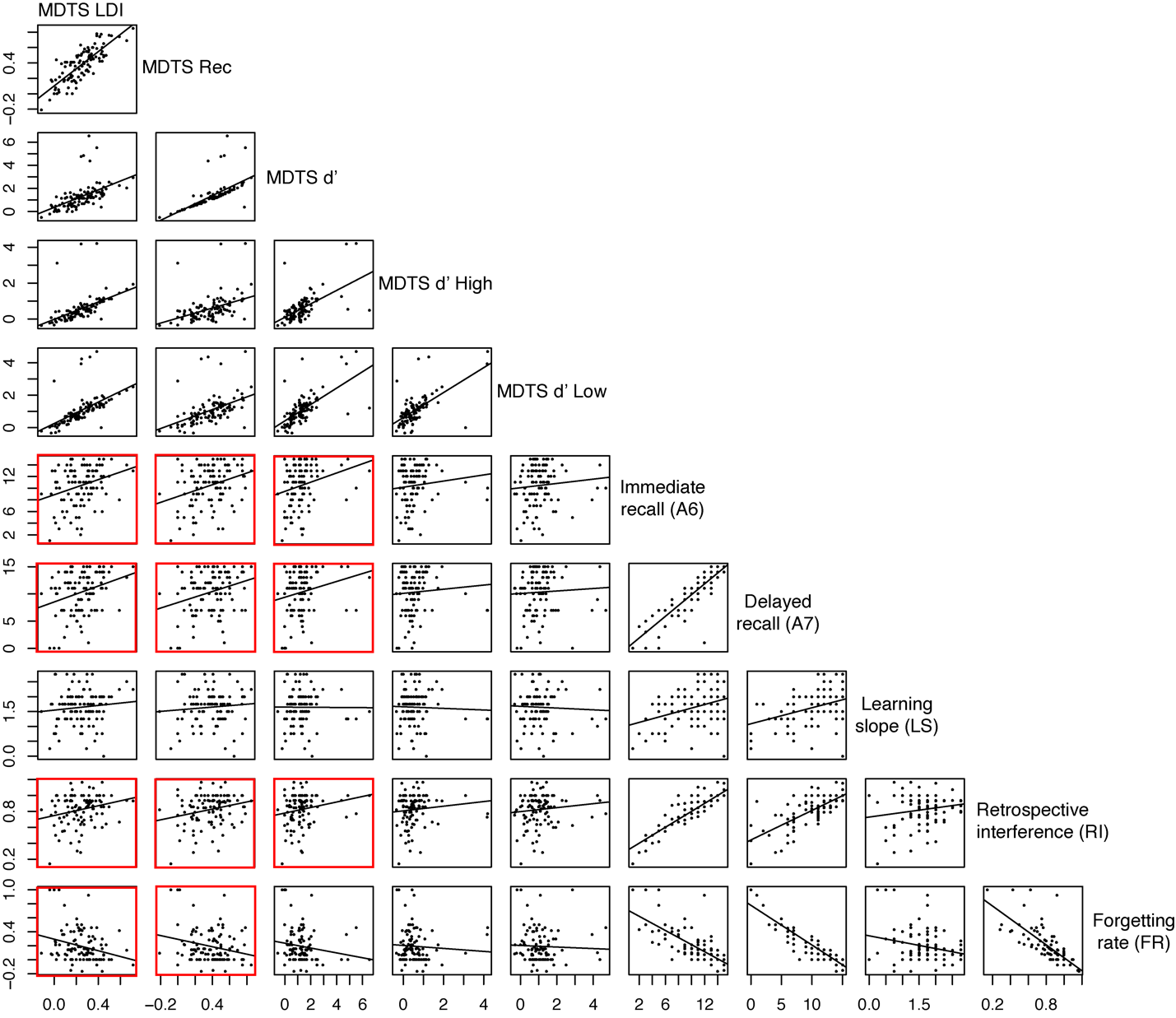


Supplemental Fig 3. Scatter plots illustrating Pearson’s correlations between performance metrics between the spatial version of the mnemonic discrimination task (MDTS) and Rey auditory verbal learning test. Significant correlations (uncorrected p < 0.05) are indicated in red. LDI = lure discrimination index, Rec = recognition, d’High = d’ for high similarity lures, d’Low = d’ for low similarity lures.


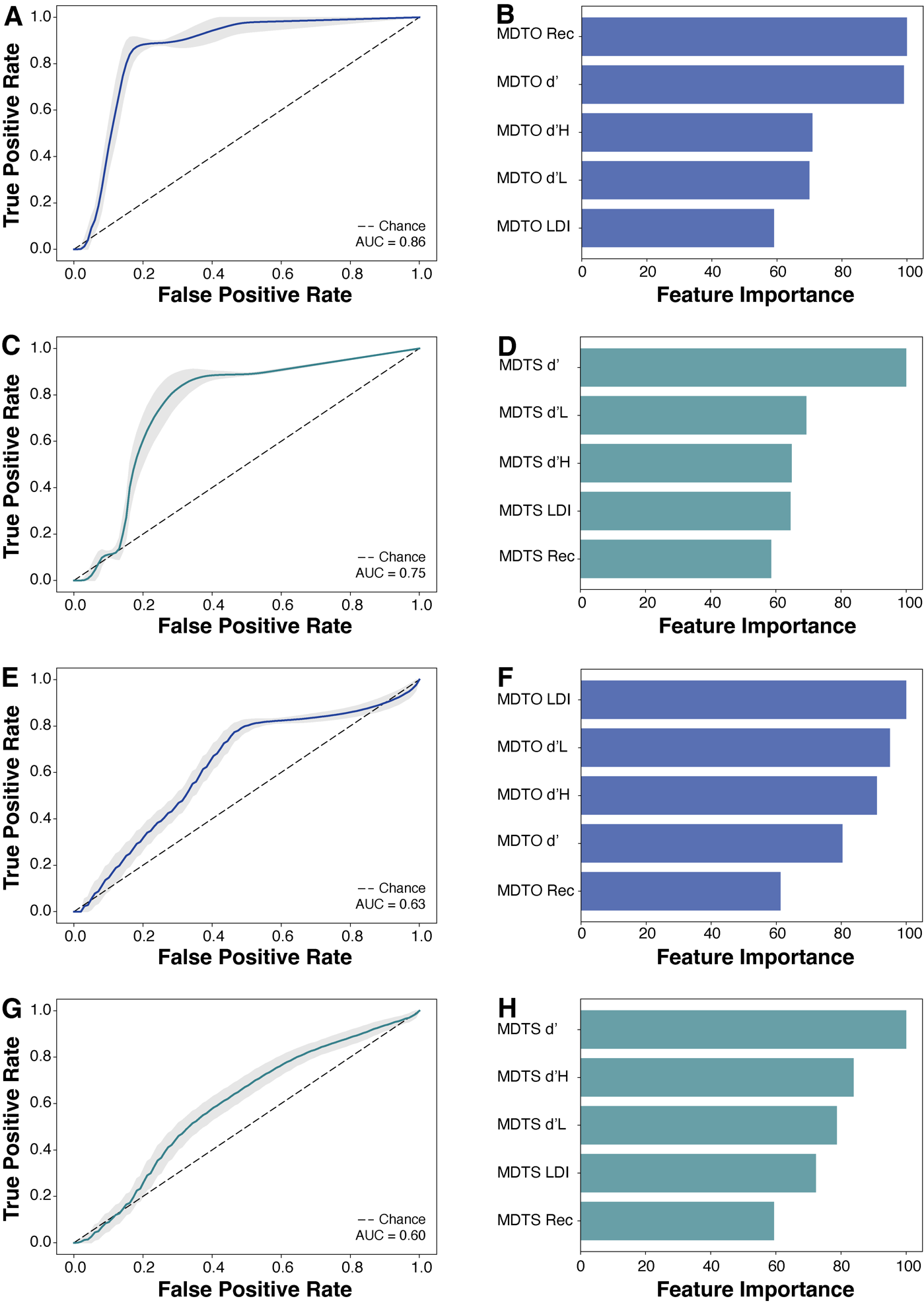


Supplemental Fig 4. Receiver operating characteristic curves and feature importance bar plots for random forest classification models using MDTO or MDTS performance metrics. For the classification of cognition, a model utilizing MDTO performance metrics (A) achieved a higher Area Under the Curve (AUC) compared to a model employing MDTS performance metrics (C). The recognition score of MDTO was found to be the most informative feature (B), while the recognition score of MDTS ranked as the least informative features (D). For the classification of amyloid status, models using MDTO performance metrics (E) and MDTS performance metrics (G) demonstrated similar AUC values. Interestingly, recognition scores were ranked the least informative features for predicting amyloid status (F, H). MDTO = object version of the mnemonic discrimination task, MDTS= spatial version of the mnemonic discrimination task, LDI = lure discrimination index, Rec = recognition, d’High = d’ for high similarity lures, d’Low = d’ for low similarity lures, Dotted line = chance.


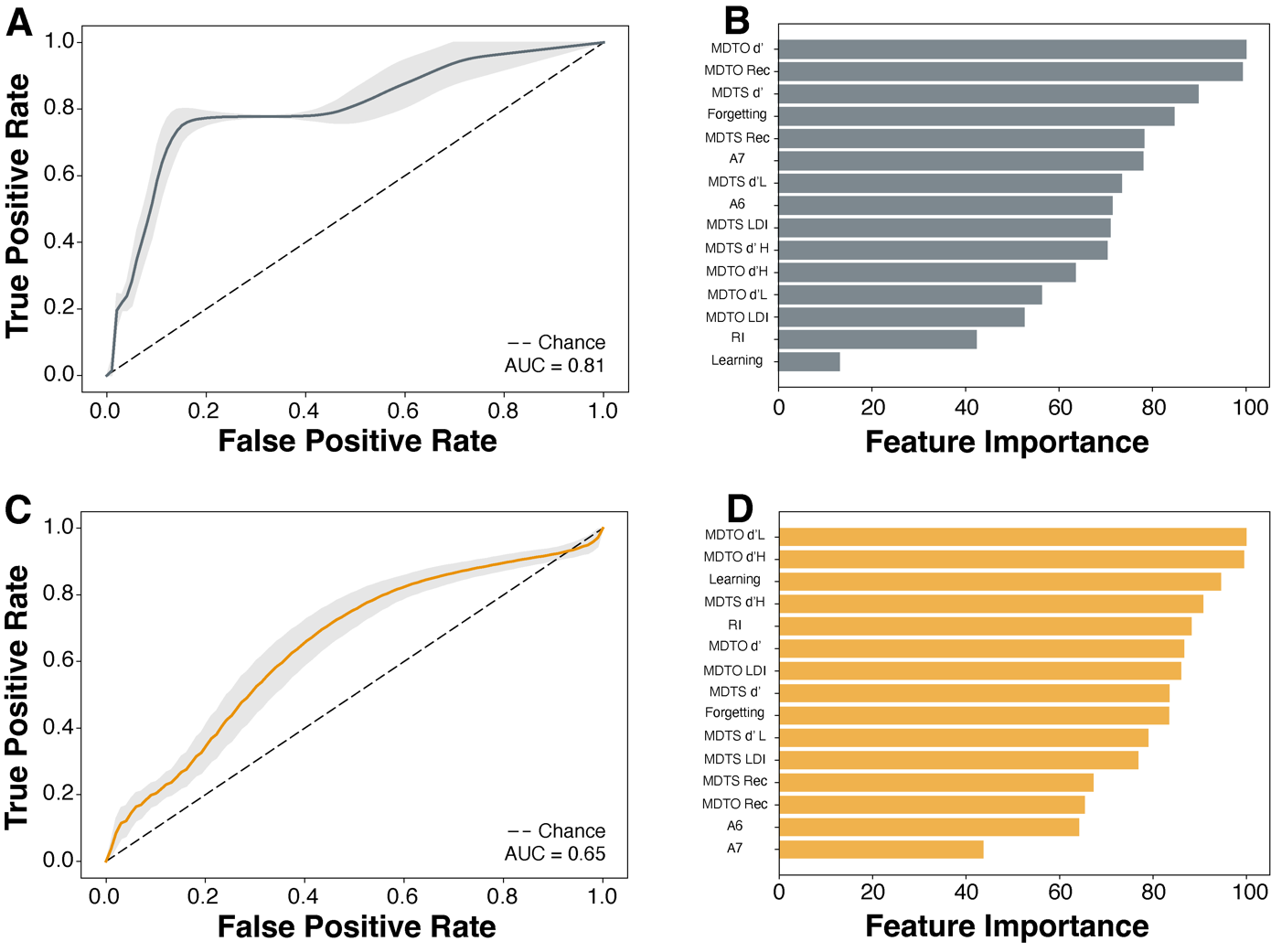


Supplemental Fig 5. Receiver operating characteristic curves and feature importance bar plots for random forest classification models using a combination of MDT and RAVLT performance metrics. For classification of cognition (A), the joint model performed as well as the model employing only the MDT performance metrics. MDT performance metrics were relatively more informative than RAVLT performance metrics (B). For amyloid classification (C), the joint model achieved a marginally higher AUC compared to the model employing the MDT performance metrics. Recall scores of RAVLT (A6 and A7) ranked as the least informative features (D). MDTO = object version of the mnemonic discrimination task, MDTS= spatial version of the mnemonic discrimination task, RAVLT = Rey auditory verbal learning test, Rec = recognition, d’High = d’ for high similarity lures, d’Low = d’ for low similarity lures, RI = retrospective interference, Learning = learning slope, Forgetting = forgetting rate, A6 = immediate recall, A7 = delayed recall, Dotted line = chance.
